# MAPK Pathway Inhibitors Attenuated Hydrogen Peroxide Induced Damage in Neural Cells

**DOI:** 10.1155/2019/5962014

**Published:** 2019-07-04

**Authors:** Zhenwei Fan, Xuan Wang, Min Zhang, Chunshan Zhao, Chunli Mei, Peng Li

**Affiliations:** ^1^Beihua University, Jilin City 132013, China; ^2^Central Hospital, Jilin City 132013, China

## Abstract

**Background:**

Oxidative stress due to reactive oxygen species plays a central role in pathophysiology of neurodegenerative diseases. Inhibition of mitogen-activated protein kinase (MAPK) cascades attenuates the oxidative induced cell stress and behaves as potential neuroprotection agent.

**Materials and Methods:**

In this study, we evaluate hydrogen peroxide induced neural cell stress and determine how different MAPK inhibitors restore the cell damage.

**Results:**

The results indicated that oxidative stress induced by neural cell damage commonly exists, and MAPK inhibitors partially and selectively attenuated the cell damage by reducing ROS production and cell apoptosis. The cultured neurons are more susceptible to hydrogen peroxide than subculture cells.

**Conclusion:**

We conclude that the essential role of different MAPK inhibitors is to attenuate the hydrogen peroxide induced neuronal cell damage. Those data broaden the implication between individual neural cells and different MAPK inhibitors and give clues for oxidative stress induced neural diseases.

## 1. Introduction

Reactive oxygen species (ROS) is a byproduct of oxygen metabolism. Generation of ROS and the activity of antioxidant defense to scavenge it result in an internal homeostasis inside the cells. However, the imbalance happens, because environmental stressors either exacerbate or impair detoxification mechanisms [1]. Abnormally high concentration of ROS can induce oxidative stress, which results in accumulated cell damage. Due to the high energy demanding and consumption activities of neural cells, they cause significant challenges to free radical scavenging. Thus, the neurons are postmitotic cells and have poor capacity to regenerate in the central nervous system (CNS) [2].

Oxidative stress–induced excessive ROS production triggers cellular damage in CNS. Hydrogen peroxide (H_2_O_2_), one of the commonly generated ROS by mitochondria, is membrane permeated and may cross cellular compartments [3]. Many macromolecules, including DNA and proteins, get damaged inside cells once excessive amount of H_2_O_2_ accumulated. The neural cells which are particularly rich in lipid content have high energy demand, and weak antioxidant capacity results in the vulnerable target of excessive ROS. In addition, ROS and the resulting oxidative stress play an essential role in apoptosis. Several key factors of apoptosis, including procaspases and cytochrome C, are released into the cytosol. Thus, there is increasing evidence showing that oxidative stress and apoptosis are closely linked physiological phenomena and are implicated in pathophysiology of CNS related disease [1, 4]. Besides apoptotic signaling, the cell cycle machinery may also be involved in oxidative stress induced DNA repair. In neurons, DNA damage and cell cycle reentry in dying postmitotic neurons give the evidence that cell cycle and apoptosis are both important components of the DNA damage response [5]. Indeed, oxidative damage is also reported in several neurodegenerative diseases, including notorious Parkinson's and Alzheimer's disease, and ROS accumulation is a threat of significant neuronal damage in neurodegenerative disorders [6–8].

In addition to cell apoptosis and cell cycle regulation, H_2_O_2_ is also able to trigger various other signaling pathways, including phosphatases and protein kinases, as well as transcription factors [9]. Here, we focus on one group of the most important proteins in the cell that responds to the accumulation of intercellular ROS, mitogen-activated protein kinases (MAPK). Several MAPK group members share structural and functional homology with each other. Intense efforts have been done to identify compounds that target different components of MAPK pathway. Small molecule inhibitors U0126, SP600125, and SB203580 (Figure 3(a)) target MEK, JNK, and p38, respectively. These inhibitors proved effective in both* in vitro* and* in vitro* models. In addition, the inhibitors were used for clinical trials for inflammatory diseases and cancer and used as pharmacological inhibitors of MAPK pathways [10, 11]. During the stimulation of H_2_O_2_ in neural cells, some of the MAPK pathways were activated and application of inhibitors effectively attenuated H_2_O_2_-induced cell damage [12–16]. However, it is unclear whether H_2_O_2_-induced cell damage is a common phenomenon among neural cells or cell line specific and how individual MAPK component inhibitor restores the cell activity, especially cell apoptosis and cell cycle changes. To this end, we compare several subculture neural cell lines, as well as primary cultured neurons, and investigate MAPK inhibitors attenuating H_2_O_2_-induced ROS production, cell apoptosis, and cell cycle changes for those cells.

In this work, we address the above question by using SH-SY5Y, PC12, and HT22 cell lines and primary neurons. The cell viability was strongly affected when neural cells were exposed to H_2_O_2_; nevertheless, the neurons were more susceptible to H_2_O_2_ decreasing cell viability. The flow cytometry results indicated that both ROS positive cells and cell apoptosis were significantly increased in all four kinds of cells, whereas the cell cycle changes were found in SH-SY5Y cells and neurons. MAPK inhibitor partially attenuated H_2_O_2_-induced damage too, but cell cycle was rarely changed. Those data argued that blocking of MAPK pathway can indeed more or less restore neural cell activity, but efficiency was diverse when individual pathway was inactive. Those results may broaden clues and implications for the treatment of oxidative stress induced neural diseases.

## 2. Materials and Methods

### 2.1. Materials and Reagents

Dulbecco's modified Eagle's medium (DMEM), Roswell Park Memorial Institute (RPMI) 1640, and fetal bovine serum (FBS) were purchased from Thermo Fisher Scientific, USA. SH-SY5Y neuroblastoma cell line (CRL226) and PC12 cell line (CRL172), derived from a transplantable rat pheochromocytoma, were both purchased from American Type Culture Collection (ATCC). The HT-22 mouse hippocampal neuronal cell line was purchased from EMD Millipore Corporation, USA. alamarBlue cell viability assay reagent was produced by Thermo Fisher Scientific, USA. 2′,7′-dichlorofluorescin diacetate (DCFDA), a fluorogenic dye that measures reactive oxygen species (ROS) activity, was purchased from Sigma-Aldrich, USA. FITC Annexin V apoptosis detection kit and cell cycle and DNA content assay kit were produced by BD Biosciences, USA. The three kinase inhibitors U0126, SP600125, and SB203580 were obtained from Calbiochem, USA. All other chemicals used were of analytical grade.

### 2.2. Cell Culture and Cell Viability Assay

SH-SY5Y and HT22 cells were cultured in DMEM medium, and PC12 cells were incubated in RPMI1640 medium. The cells were supplied with 10% FBS (v/v), and where appropriate, 100 U/ml penicillin and 100 *μ*g/ml streptomycin were added to the medium. A humidified atmosphere incubator containing 5% CO_2_ was used to maintain regular cell growth at 37°C. The medium was replaced every two days [17]. The neuron cells were cultured as described before [18]. Briefly, we isolated and cultured pyramidal neurons from the early postnatal (P0-P1) mouse cortex and then cultured them on poly-L-lysine coated glass substrates. Neurons can be maintained up to four weeks according to the procedure.

The cell viability was measured using alamarBlue cell viability assay reagent following the manufacturer's instructions. Briefly, roughly 10,000 cells or primary neurons were cultured into the 96-well plates. When the cell intensity reached 70%-90% coverage, additional H_2_O_2_ was added to the medium to make a final concentration of 1000 *μ*M, followed by twofold serial dilution. After dilution, 100 *μ*l of medium was left in individual well. alamarBlue reagent was added directly to each well after 12 or 24 hours of incubation, and plates were incubated at 37°C for additional four hours in the dark, which allows the cells to convert resazurin to resorufin. Finally, fluorescence signal was read using excitation wavelength at 570, and emission peak at 585 nm was used to determine relative cell viability. A control sample without cells seeded was used as the blank, and cells without H_2_O_2_ added were used as positive control. All the tested concentrations were performed three times with a minimum of six replicates. Results were evaluated by normalized fluorescent signal to positive control group and plotting relative cell viability versus H_2_O_2_ concentration. The concentration of H_2_O_2_ that gives half-maximal response (EC50) was then fitted to a Hill equation, as appropriate [19].

### 2.3. Flow Cytometry Analysis

Cellular ROS production was determined by the cell permeant reagent DCFDA. Cells were washed or digested by 0.25 mg/ml trypsin and then treated with 10 *μ*M DCFDA in medium at 37°C for 30 min in the dark. After staining, cells were passed through a 40 *μ*m cell strainer. Without washing, aliquot cells were subjected to flow cytometry equipped with the Cell Quest software; at least 10,000 cells were analyzed at excitation and emission wavelengths of 485 nm and 535 nm, respectively.

Propidium iodide (PI) and Annexin V combination staining is commonly used to determine if cells are apoptotic or necrotic through the differences between plasma membrane integrity and permeability [20, 21]. Here, we use flow cytometry-based assay to identify the effect by appropriate EC50 concentration of H_2_O_2_ in individual cells. Harvested cells were washed twice with cold PBS and then resuspended at the concentration of about 1 × 10^6^ cells/ml in binding buffer provided by the kit. 5 *μ*l of FITC Annexin V and PI each were used per test, with gently stirring to mix the cells and incubating for 15 min at room temperature in the dark. A minimum o f10,000 cells were analyzed within 1 hour.

Based on cellular DNA content by staining with PI, a DNA-specific stain, we determined the percentage of the subpopulation in G0/G1, S, and G2/M by flow cytometry [22]. During the cell cycle, the DNA content increases as new DNA is replicated. As such, cells in different stages of the cell cycle are tightly regulated with different DNA contents. The intensity of PI fluorescence inside the cell is directly proportional to the DNA content. Cells were resuspend in PBS at the concentration of about 1 × 10^6^ cells/ml and stirred gently several times in order to minimize cell aggregation. Cells were fixed by 70% ethanol and kept on ice for at least 2 hours. After a brief washing by PBS twice, cell pellet was dissolved in 1 ml of PI staining solution in the dark at room temperature for 25 min. The cells were transferred to flow cytometer and fluorescence was measured within 1 hour.

### 2.4. Statistics

The results were expressed as the average ±standard deviation (SD). Unless otherwise indicated, at least three biological repeats were performed. Student's *t*-test was used to evaluate the significance. The significance level cutoff was 0.05 and 0.01. *P* < 0.05 is considered significant (#); *P* < 0.01 is considered highly significant (*∗*).

## 3. Results

### 3.1. H_2_O_2_-Induced Cell Viability Damage in Neural Cells

**Figure 1:**
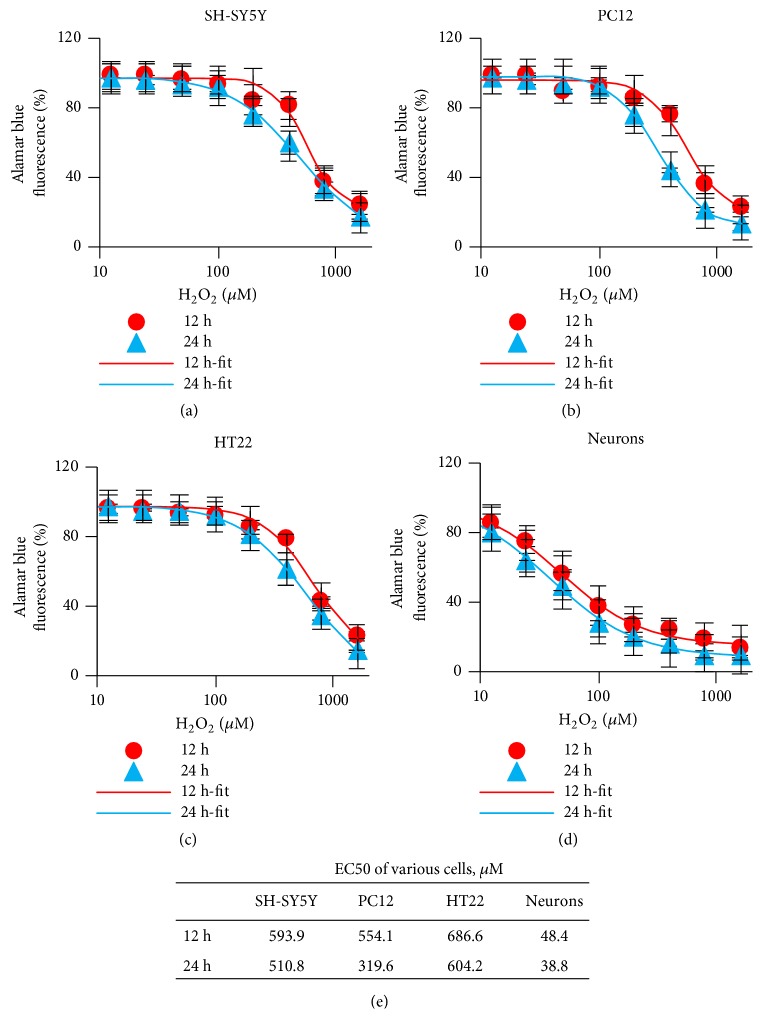
H_2_O_2_-induced cell viability changes in various cells for 12 and 24 hours. (a) SH-SY5Y. (b) PC12. (c) HT22. (d) Neuron cell viability was determined by alamarBlue reagent assay and expressed as indicated concentration of H_2_O_2_. (e) The EC50 of H_2_O_2_ was calculated and shown in the table (n=6).

ROS production in normal metabolism of oxygen plays an important role in cell signaling. However, accumulation of excess ROS is a potent inducer of dysfunction in CNS, which results in oxidative damage and various neurodegenerative disorders. Chronically elevated levels of H_2_O_2_ have been implicated in cell viability in multiple neuronal cells [23, 24]. To determine the H_2_O_2_ toxicity on neural cells, alamarBlue reagent was used to assess cell viability. Resazurin, a nontoxic, membrane permeable nonfluorescent compound, is the active ingredient in this assay. Living cells continuously convert resazurin to resorufin, a red color and highly fluorescent compound [25]. Here, the cell viability of SH-SY5Y, PC12, HT22, and primary neurons was evaluated after 12 and 24 hours. The cells were exposed to different concentrations of H_2_O_2_ when cell intensity covered 70%-90% of growth area. Cell viability of all tested cells markedly decreased, which was followed by incubation with H_2_O_2_ with dose-dependent manner (Figures 1(a)–1(d)). The cell viability of SH-SY5Y, PC12, and HT22 cells was not affected when supplied with up to 100 *μ*M H_2_O_2_, whereas the primary neuron cells were more susceptible. Supply with 100 *μ*M H_2_O_2_ resulted in a decrease to about 40% relative cell viability. When cells were exposed to 1000 *μ*M H_2_O_2_ of high concentration, the cell viability was strongly decreased in all tested cells. It indicated that H_2_O_2_ -induced cell damage existed both in subculture and primary cultured cells. Notably, the cells exposed to H_2_O_2_ at two time points, 12 hours and 24 hours, did not result in significant difference. This suggests the damage of H_2_O_2_ was accumulated within less period. Further, the EC50 of H_2_O_2_ was calculated (Figure 1(e)). Indeed, the EC50 of H_2_O_2_ for SH-SY5Y, PC12, and HT22 at 12 hours was 593.9, 554.1, and 686.6 *μ*M, respectively. There is a dramatically reduction in primary neurons, of which, the EC50 of H_2_O_2_ was 48.4 *μ*M. The individual EC50 at 24 hours was a little lower than the one at 12 hours. Thus, the relative concentration of H_2_O_2_ close to EC50 was used for the following assays. 600 *μ*M H_2_O_2_ was used for subculture cell lines, while 50 *μ*M H_2_O_2_ was selected for primary neurons. Those results indicated that neural cell viability was strongly affected when cells were exposed to H_2_O_2_. The neurons were more sensitive to H_2_O_2_ damage.

### 3.2. H_2_O_2_-Induced ROS Production, Cell Apoptosis, and Cell Cycle Changes

The oxidative stress caused by H_2_O_2_ has been implicated in the pathophysiology of various neurological disorders. It usually affects intercellular ROS production, triggers mechanism in neuronal cells, and leads to abnormal phonotypical changes [26]. To identify the effect of H_2_O_2_ on ROS production, the intracellular ROS was determined by DCFDA-based flow cytometry assay. The cell permeant reagent DCFDA is deacetylated to a nonfluorescent compound and further oxidized to a highly fluorescent compound 2′,7′-dichlorofluorescein (DCF) which is proportionally increased with ROS production [27]. Here, the relative EC50 concentration of H_2_O_2_ was used to compare the ROS production in different neural cells (Figures 1(a) and 1(b)). The ratio of ROS positive cells was lower than 4%, where no H_2_O_2_ was added in all cells. It indicated the functional balance of ROS and cell ability to detoxify the resulting damage. The results showed that H_2_O_2_ treatment significantly increased the production of ROS, suggesting that H_2_O_2_ should be radically most responsible for oxidative neuronal damage.

**Figure 2:**
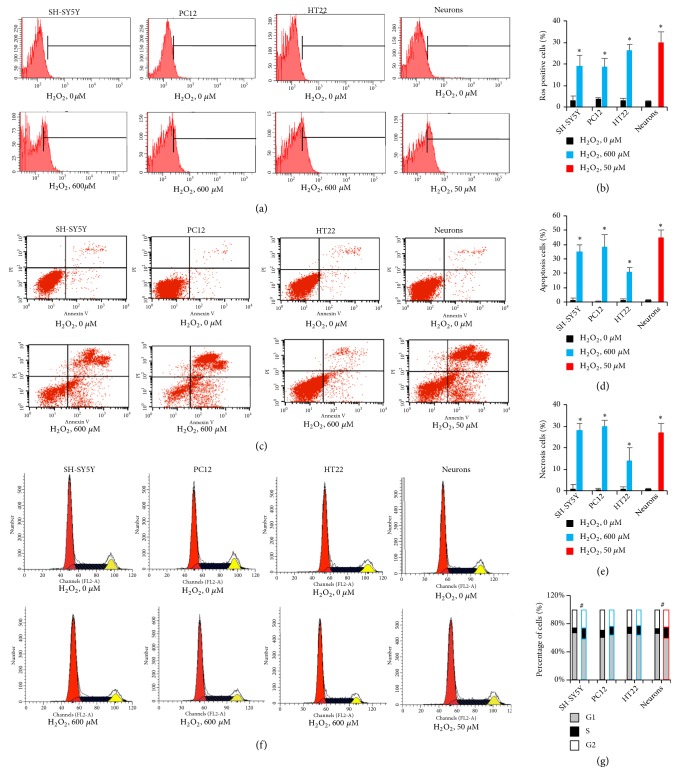
H_2_O_2_-induced ROS production, cell apoptosis, and cell cycle change by flow cytometry. (a) The ROS positive cells were determined using ROS-sensitive fluorometric probe. (c) The cell apoptosis and necrosis were measured by Annexin V and PI straining. (e) The cell cycle change was calculated by PI straining. The percentage of cell portions was further shown in subfigures (b) ROS production, (d) cell apoptosis and necrosis, and (f) cell cycle changes, respectively (n=10,000 cells in all experiments).

The ROS-induced oxidative stress in neuronal cells triggers various cell programs. The ensuing dysfunction of mitochondria in neural cells has been demonstrated in relation to neurological diseases. Following oxidative stress, several proapoptotic molecules are activated, leading to intrinsic apoptosis [28]. To this end, we determine the cell apoptosis or necrosis, by taking advantage of the flow cytometry assay for double staining Annexin V and PI to distinguish these two cell processes. Addition of H_2_O_2_ to neural cells resulted in both cell apoptosis and necrosis (Figures 2(c)–2(e)). The results revealed that H_2_O_2_ induced a significant increase in programmed cell death, as well as mechanical damage.

We next investigated how H_2_O_2_-induced ROS production modulates cell cycle. The 70% ethanol fixed cells were used to allow PI permeabilization to cells. This dye will bind in proportion to the amount of DNA present in the cell [29]. The percentage of cells in S phase increased compared with control group after incubation with H_2_O_2_ for 12 hours in SH-SY5Y and neurons cells (Figures 2(f) and 2(g)). Accordingly, the percentage of cells in G2 phase decreased. No significant cell cycle changes were found in PC12 and HT22 cells. Those results suggested that H_2_O_2_ induces moderate cell cycle arrest in neural cell, and different mechanism exists in neural cell lines to compensate for the H_2_O_2_ damage. Taken together, these findings indicated that H_2_O_2_ leads to neurological disorders where neuronal oxidative stress contributes.

### 3.3. MAPK Pathway Inhibitors Attenuated H_2_O_2_-Induced Cell Damage

**Figure 3:**
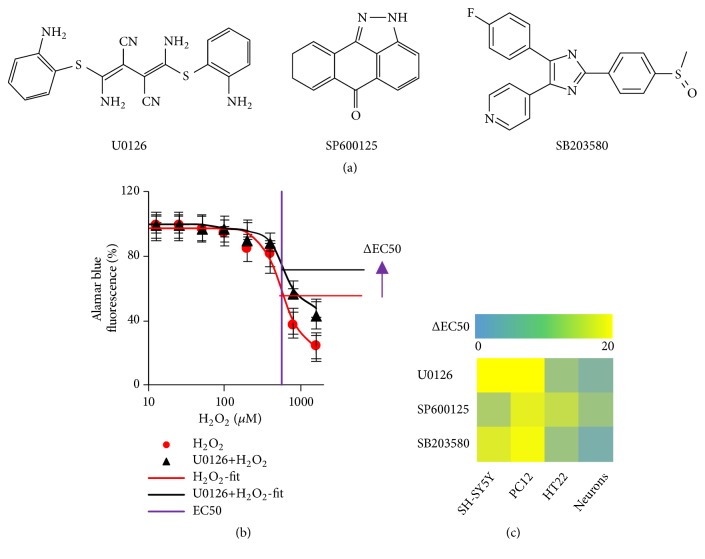
H_2_O_2_-induced cell viability was partially restored by blocking MAPK pathway. (a) Chemical structure of MAPK pathway inhibitors, including SP600125 and SB203580, targeting MEK, JNK, and p38, respectively. (b) The EC50 increased when cells were supplied with U0126 inhibitor. (c) Relative increase in EC50 by heat map. H_2_O_2_-induced cell viability was partially restored by individual MAPK inhibitor.

Oxidative stress induced by excess ROS has been implicated in pathologic processes associated with neurodegenerative diseases. In neuronal cells, various intracellular signaling pathways strictly control cell function. H_2_O_2_ causes activation of the MAPK pathway and this is followed by reducing the damage of neural cell [30]. Here, we investigated how individual MAPK inhibitors block effects of H_2_O_2_-induced neural cell damage. Three well-known MAPK inhibitors that had highly different structures were selected (Figure 3(a)). The inhibitors of MAPK, namely, MEK inhibitor U0126, JNK inhibitor SP600125, and p38 inhibitor SB203580, were added to the corresponding wells. We used 10 *μ*M of those inhibitors, which proved effective without affecting cell viability before [31, 32]. H_2_O_2_ with or without inhibitor was added to the wells, and the cells were cultured for another 12 h. The ΔEC50 was calculated and plotted as the heat map (Figures 3(b) and 3(c)). Those inhibitors selectively attenuated H_2_O_2_-induced cell damage by increasing their cell viability. Interestingly, the cell viability of SH-SY5Y and PC12 was increased by supplying all three inhibitors, while the neurons cell viability was rarely increased. These results suggest that H_2_O_2_-induced oxidative stress was strongly associated with activation of MAPK. Inhibition of MAPK downstream pathways led to increasing the cell viability.

### 3.4. MAPK Pathway Inhibitors Attenuated H_2_O_2_-Induced ROS Production

**Figure 4:**
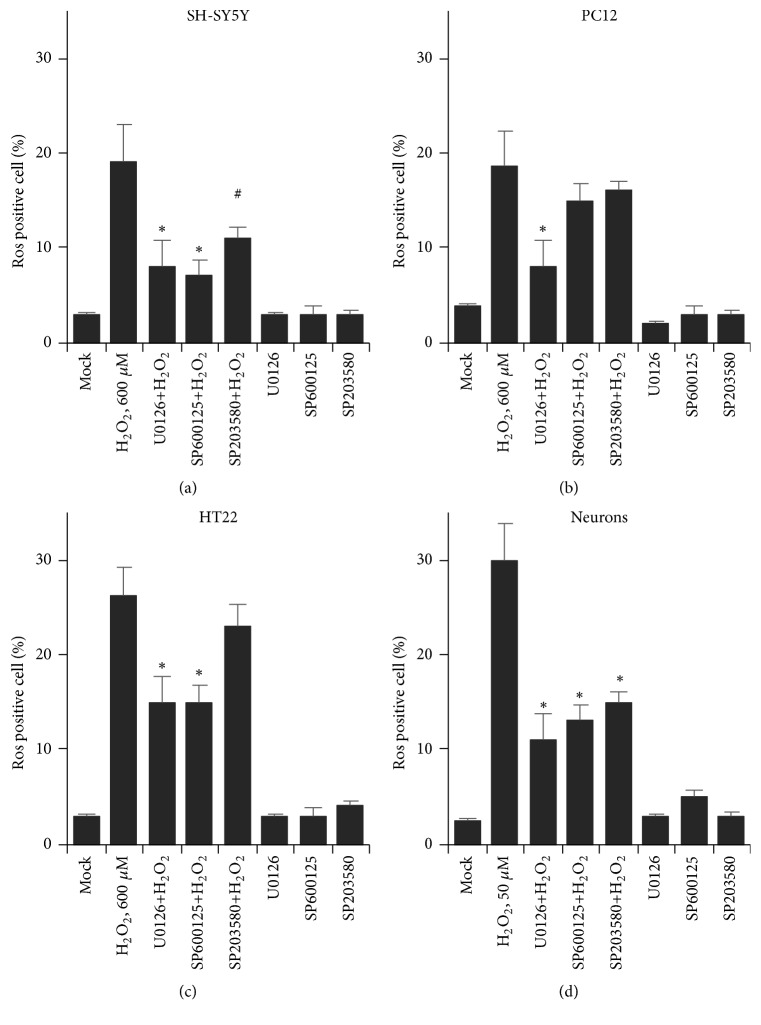
H_2_O_2_-induced ROS positive cells were selectively decreased by MAPK inhibitor. (a) SH-SY5Y. (b) PC12. (c) HT22. (d) Neurons ROS positive cells were determined by flow cytometry with either a combination of inhibitor and H_2_O_2_ or the inhibitor itself (n=10,000 cells). # indicates *P* < 0.05; *∗* indicates *P* < 0.01.

Next, we determined whether ERK, JNK, and p38 activation were involved in H_2_O_2_-mediated oxidative stress. 10 *μ*M of U0126, SP600125, and SB203580 was added to the corresponding wells. The ROS positive cells were measured by flow cytometry, and the data indicated that ROS production was not affected in all cells by adding those inhibitors separately (Figures 4(a)–4(d)). We took appropriate EC50 concentration of H_2_O_2_ to individual cells by supplying them with MAPK pathway inhibitors. When supplied with H_2_O_2_, the ROS positive cells increased dramatically in all the four kinds. Blocking any of MEK, JNK, or p38 pathway resulted in attenuated ROS production in SH-S5Y5 cell and primary neurons (Figures 4(a) and 4(d)). However, the percentage of PC12 ROS positive cells could only be rescued by MEK inhibitor U0126 (Figure 4(b)). In addition, both MEK inhibitor U0126 and JNK inhibitor SP600125 partially inhibit intercellular ROS production, leading to the significant decrease in ROS positive cells (Figure 4(c)). Notably, the MEK inhibitor could partially restore H_2_O_2_-mediated ROS production in all tested cells. These results demonstrate that oxidative stress-mediated MAPK activation plays an essential role in neural cells ROS production. Our findings suggested that inhibition of MEK activation or maybe inactivation of a combination of MAPK pathways might be a potential strategy for oxidative stress induced neural cell damage.

### 3.5. MAPK Pathway Inhibitors Attenuated H_2_O_2_-Induced Neural Apoptosis

**Figure 5:**
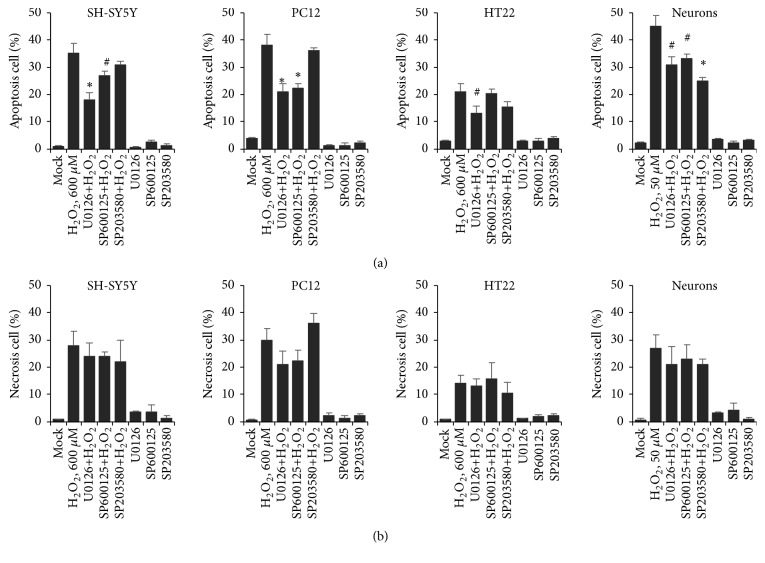
H_2_O_2_-induced cell apoptosis was selectively attenuated by MAPK inhibitor. (a) Cell apoptosis and (b) necrosis were measured by Annexin V and PI straining. The percentage of those two events was shown in histogram (n=10,000 cells). # indicates <0.05; *∗* indicates <0.01.

To determine to how MAPK pathway inhibitors affect H_2_O_2_-induced neural apoptosis and necrosis, we performed Annexin V and PI double straining assay to neural cells with addition of single inhibitor, and the results were measured by flow cytometry. The data showed that EC50 concentration H_2_O_2_ induced both cell apoptosis and cell necrosis, whereas these two events were not affected when any single inhibitor was added (Figures 5(a) and 5(b)). The proportion of cell apoptosis was partially reduced when inhibitor was added, and supply with MEK inhibitor U0126 led to decreased cell apoptosis in all groups. However, the cell necrosis was hardly affected by blocking any of the MAPK involved pathways. By using the neural cell model, we concluded here that apoptotic targets are potently activated by AMPK. Inactive AMPK pathway, more or less, resulted in decreasing cell apoptosis. Importantly, these events occur without affecting cell necrosis at all.

### 3.6. MAPK Pathway Inhibitors Hardly Restore H_2_O_2_-Induced Cell Cycle Arrest

**Figure 6:**
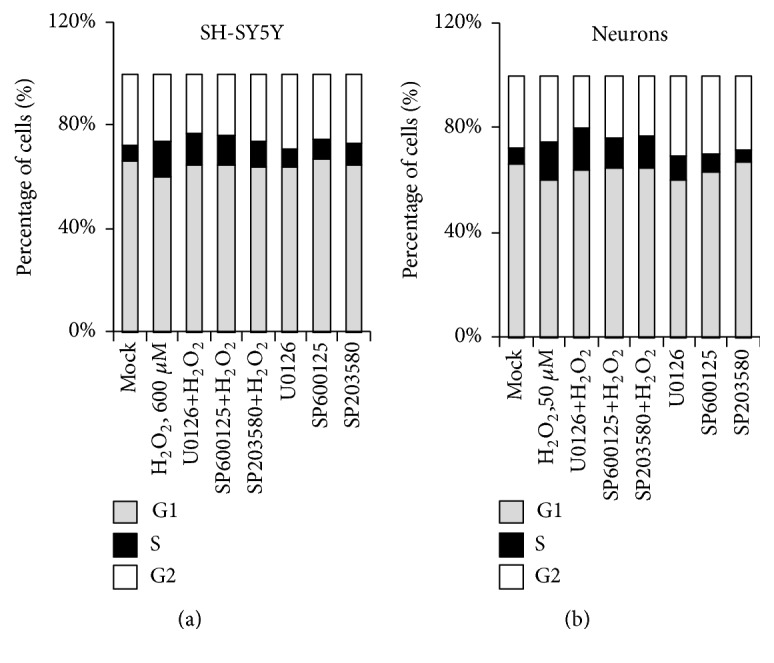
H_2_O_2_-induced cell cycle changes were not affected by MAPK inhibitor. (a) SH-SY5Y and (b) neurons ROS cell cycle changes were determined by PI straining (n=10,000 cells).

Both apoptosis and cell cycle are highly conserved mechanisms. Eukaryotic cells need to adapt the changing stress through the coupling of the cell cycle and programmed cell death by using or controlling a shared set of factors [33, 34]. Since cell apoptosis was restored by inhibiting AMPK pathways when supplying cells with H_2_O_2_, we next determined whether MAPK inhibitors affect H_2_O_2_-induced cell cycle arrest. It has been shown that the S phase increased after incubation with H_2_O in SH-SY5Y and neurons cells (Figure 2(g)). Therefore, we took those two cell models and all three inhibitors U0126, SP600125, and SB203580 were tested by incubation with the cells alone or adding H_2_O_2_ simultaneously. Interestingly, none of MAPK pathway inhibitors could restore H_2_O_2_-induced cell cycle arrest (Figures 6(a) and 6(b)). The results suggested regulation of cell cycle arrest and cell apoptosis can share different mechanism in H_2_O_2_-induced neural cell damage.

## 4. Discussion

ROS production in neural organisms is the normal oxygen metabolism, which is tightly controlled by endogenous respiratory chain and enzymatic reactions. However, high concentrations of ROS, as H_2_O_2_, can damage intercellular macromolecules like DNA, proteins, and lipids, which results in enormous cell damage [35]. Therefore, oxidative stress induced damage to CNS has a strong potential to negatively impact its normal functions, mainly in neurodegenerative disorders disease [36, 37].

In this study, the* in vitro* model results indicated that H_2_O_2_ induced significant damage to neural cells, including decreasing the cell viability, cellular ROS production, cell apoptosis, and cell cycle changes. The MAPK inhibitors selected in this study are essential. Frist, the MAPK is a key energy sensor and regulator. The interplay between MAPK and ROS is complex in CNS. Inactive MAPK pathway showed anti-cell damage effects, through the suppression of ROS by modulating the level or activity of multiple factors [38]. Second, MEK, JNK, and p38 pathways were well studied before and are strongly associated with ROS production. U0126, SP600125, and SB203580 inhibitors target MEK, JNK, and p38, respectively, and could effetely block the activation of their target. Therefore, supplying those inhibitors potentially acts against ROS damage. On the other hand, when the neuronal cells were supplied with the H_2_O_2_, a strong oxidative stress was generated. Cell physiology changes significantly to adapt the change. Many macromolecules get damaged inside cells once excessive amount of H_2_O_2_ accumulated. Accumulating evidence has shown that cell proliferation was regulated and achieved, at least partially, by coupling cell cycle progression and programmed cell death. However, by blocking MAPK pathway, rescuing H_2_O_2_-induced neural cell damage was highly diverse. Thus, the cell apoptosis and cell cycle changes were not well linked by MAPK pathway.

## 5. Conclusions

In this study, we compare the cell damage of H_2_O_2_ against both subculture neural cells and primary culture neurons, and MAPK pathway inhibitors attenuate the damage. The results indicated that neurons were more susceptible, whereas the EC50 was about 10-fold less than other neural cells. ROS production and cell apoptosis were severely damaged when neural cells were supplied with H_2_O_2_; however, cell cycle change was moderate. Inhibition of MAPK pathway by adding ERK, JNK, or p38 inhibitor resulted in attenuating cell damage. The evidence supports the fact that exogenous addition of H_2_O_2_, one of the ROS, was an important factor involved in MAPK pathways. The hierarchy of those events broaden our understanding of the role of MAPK in ROS signaling among neural cells.

## Data Availability

The data used to support the findings of this study are included within the article.
